# Social Determinants of Cancer Risk Among American Indian and Alaska Native Populations: An Evidence Review and Map

**DOI:** 10.1089/heq.2022.0097

**Published:** 2022-09-21

**Authors:** Stephanie C. Melkonian, Jolie Crowder, Emily E. Adam, Mary C. White, Lucy A. Peipins

**Affiliations:** ^1^ Division of Cancer Prevention and Control, Centers for Disease Control and Prevention, Albuquerque, New Mexico, USA.; ^2^ International Association for Indigenous Aging, Silver Spring, Maryland, USA.; ^3^ Division of Cancer Prevention and Control, Centers for Disease Control and Prevention, Atlanta, Georgia, USA.; ^4^ Oak Ridge Institute for Science and Education, Oak Ridge, Tennessee, USA.

**Keywords:** American Indian and Alaska Native, health equity, social determinants of health

## Abstract

**Objectives:**

To explore current literature on social determinants of health (SDOH) and cancer among American Indian and Alaska Native (AI/AN) populations.

**Methods:**

We searched Ovid MEDLINE^®^, CINAHL, and PsycINFO databases for articles published during 2000 to 2020, which included terms for SDOH and cancer occurrence in AI/AN populations. We derived the data extraction elements from the PROGRESS-Plus framework. The Preferred Reporting Items for Systematic Reviews and Meta-Analyses (PRISMA)-Equity extension guided the evidence map.

**Results:**

From 2180 screened articles, 297 were included. Most were observational (93.9%), employed a cross-sectional design (83.2%), were categorized as cancer occurrence and surveillance research (62%), and included no cancer-related risk factors (70.7%). Race, gender, and place were the most frequently included PROGRESS-Plus categories. Religion, relationship features, and characteristics of discrimination were least common. Only 12% of articles mentioned historical/current trauma or historical context.

**Conclusions:**

Gaps exist in our understanding of SDOH as drivers of cancer disparities in AI/AN populations. Future studies in health equity science may incorporate historical and cultural factors into SDOH frameworks tailored for AI/AN populations.

## Introduction

The disproportionate impact of preventable diseases among American Indian and Alaska Native (AI/AN) populations in the United States is well documented.^1–3^ Persistent inequities in resources and opportunities contribute to significant disparities^2,4,5^ in cancer incidence and morbidity across AI/AN populations and between AI/AN populations and other racial and ethnic populations.^1,6,7^ Cancer incidence varies by geographic region across AI/AN populations for many cancer types.^1,6,7^ Despite efforts to address individual-level risk factors such as screening utilization and recreational smoking, disparities persist in the incidence of certain cancers.^6^ Population-level environmental, social, and structural factors strongly influence individual health and play a foundational role in public health.^8,9^

This article uses a framework for the organization of social and structural factors and is guided by definitions from Healthy People 2030,^10^ the World Health Organization,^11^ and the National Academies of Science, Engineering, and Medicine.^12^ According to Healthy People 2030, social determinants of health (SDOH) are “the conditions in the environments where people live, learn, work, worship, and play” that affect a wide range of health and quality of life outcomes and risks.^10^ SDOH domains include education access and quality, economic stability, neighborhood and built environments, social and community context, and access to quality health care.^10^

SDOH arise from both historical and contemporary structural inequities, defined by a committee of the National Academies of Sciences, Engineering, and Medicine as the “systematic disadvantage of one social group compared to others,”^12^ driven by factors such as racism, sexism, ageism, classism, and other forms of social exclusion or social marginalization. The complex distribution of power and resources at local, national, and global levels, and the mechanisms by which they are organized along lines of group identity, are the causal forces that produce inequities.^12,13^ Tailored approaches to addressing these root causes and related SDOH require an understanding of the unique drivers of health disparities in different populations.^3,12^

Solutions aimed at addressing SDOH and the associated systems, policies, and practices that create inequities present the greatest opportunity to reduce cancer disparities among AI/AN populations. The development of an equity-focused framework for SDOH, specifically for the drivers of cancer disparities, is a precursor to this work. To date, there has been no systematic assessment of SDOH with regard to cancer occurrence among AI/AN populations.

### Historical context

AI/AN persons have been the subject of centuries of systemic racism and discriminatory policies and practices. These include colonization, dispossession from homelands through forced removal and relocation, forced attendance at residential boarding schools, and harmful policies aimed at assimilation, acculturation, and termination of sovereign rights and citizenship.^3^ Early genocidal practices nearly eradicated AI/AN communities. Multiple researchers have attributed the origins of modern social adversities to historical traumas and atrocities wrought upon AI/AN people spanning generations.^14–16^ While this association requires further investigation, emerging studies suggest that this historical trauma may have led to the acquisition of intergenerational stress.^3,14,17^

Adverse experiences such as poverty, racism, domestic violence, and unintentional injuries can lead to poor physical and mental health outcomes among AI/AN persons.^2,3^ Poor physical and mental health outcomes can result from lack of access to care, access to healthy foods, built environment, and opportunities related to educational attainment and employment.^2,14^ Researchers have established that ongoing health disparities among AI/AN populations in the United States^3^ persist in virtually all areas of health, including chronic disease,^18^ cancer,^6^ and most recently, the COVID-19 pandemic.^19^

AI/AN populations and communities continue to find strength through tribal culture and traditional practices.^20,21^ This is despite policies designed to disrupt cultural practices and ways of being that have contributed to health inequities.^4,14^ Many current public health research methods and datasets are not designed to account for the geographic, cultural, structural, and linguistic intertribal variations of the 574 currently federally recognized AI/AN tribes.^22^ Furthermore, the unique characteristics and histories of different AI/AN communities can be masked when researchers aggregate AI/AN persons into a single group.^5,7^

### Study purpose

The purpose of this systematic search and evidence map review is to quantify and characterize the current evidence addressing SDOH and cancer occurrence among AI/AN populations.

## Methods

We used the Preferred Reporting Items for Systematic Reviews and Meta-Analyses (PRISMA)-Equity extension to guide our systematic search and evidence mapping process.^23^ The Equity extension recommends using the “PROGRESS-Plus” categories to aid in the classification of equity in reviews. PROGRESS categories, constructed in 2003, include the place of residence, race or ethnicity, occupation, gender, religion, education, socioeconomic status (SES), and social capital (e.g., marital status or family support).^24^ Additional categories added later (Plus) include personal characteristics associated with discrimination, such as disability or sexual orientation, features of relationships (i.e., family history of cancer, adverse childhood experiences), and time-dependent relationships (i.e., age at cancer diagnosis), which are also included in this article.^25^ Supplementary Data includes additional examples.

Use of the term “race“to describe AI/AN persons and diverse tribal citizenry as a single whole has been characterized as historically problematic.^8,26^ Race is widely recognized as a socially constructed categorization based largely on markers of difference such as phenotype or behavior; race differs from ethnicity, genetic ancestry, or biology.^8,26^ For members of federally recognized tribes, AI/AN race is determined based on eligibility for federal benefits.^26^ Efforts are underway to replace the use of race with other terms, including ethnicity, tribal affiliation, political entity, and ancestral identity.^27^ The term “race” is used in this article to maintain fidelity to the PROGRESS-Plus framework. For the purposes of this review, the term “race” for AI/AN persons also includes ethnicity, language, culture, and ancestry.

### Eligibility criteria

Eligible studies included AI/AN groups either as the sole study population, as a comparator within AI/AN race, or between AI/AN groups and other racial groups. A cancer outcome variable related to disease occurrence was required for inclusion (i.e., cancer incidence, cancer screening, cancer risk, community-based intervention related to cancer risk reduction, or cancer screening). Intervention studies focused on health providers were excluded to maintain focus on individual- and community-level efforts. Also excluded were intervention studies that did not include cancer incidence or screening as an outcome. Quantitative and mixed-methods studies were included. Full exclusion criteria can be found in the Supplementary Data.

### Search strategy and study selection

We conducted a Boolean search string in our query based on previous studies of social determinants of cancer.^28–31^ See Supplementary Data for a sample search string. We conducted the search in Ovid MEDLINE^®^, CINAHL, and PsycINFO for articles published from January 2000 to May 2020. Articles were initially reviewed and duplicates removed by one researcher (J.C.) using Microsoft Excel, and then migrated to Covidence systematic review software, where 33 additional duplicates were automatically removed.^32^ Risk of bias in study results and other indicators of article quality were outside the scope of this review.

### Title and abstract review

Two research assistants (J.B. and S.Z.) independently reviewed titles and abstracts to screen and tag outcome categories. The outcome categories used were cancer occurrence and surveillance (these articles include only studies of cancer incidence or prevalence); early detection/screening; etiology/risk factors; and screening interventions. While other types of intervention studies were eligible, only screening-related interventions were included in the final review, as reflected in the outcome category. Discrepancies were resolved by team consensus or by the project leads (J.C. and S.C.M.).

A pilot review of a subset of 61 article titles and abstracts tested for inter-rater reliability produced a percent agreement of 92% and a Cohen's kappa of 0.82. Final percent agreement was 94% with a Cohen's kappa of 0.87. Six hundred seventy articles remained for full-text review. Feedback from the team and from a PROGRESS-Plus framework subject matter expert (J. Petkovic, personal communications, November 12, 2020) included a suggestion to modify the exclusion criteria to focus on articles specifically related to cancer prevention and screening. The International Cancer Research Partnership Common Scientific Outline guided cancer and research-type categorizations.^33^

### Data extraction

Supplementary Data shows data extraction criteria and definitions. The purpose of data extraction was to measure the inclusion of health equity themes according to PROGRESS-Plus categories. Inclusion of these categories was recorded as yes/no. In addition, free text entries of PROGRESS-Plus subcategories/themes and information on cancer risk factors as study variables were extracted. Researchers separately assessed whether historical trauma, current trauma, or historical and current trauma were discussed (yes/no) and whether the Institutional Review Board (IRB) or tribal review or approval processes were mentioned or addressed (yes/no). Tribal sovereignty extends to regulatory rights that have implications for research, including IRB requirements, on tribally governed lands.^34^

Data were extracted by two review teams (S.C.M. and E.E.A., J.C. and J.B.). The extraction tool was piloted by both teams, and subsequent revisions were agreed upon by consensus. Teams conducted data extraction in batches with regular meetings to discuss, review, and achieve consensus on the extraction variables. Any remaining uncertainty was resolved by a third senior reviewer as needed. Finally, one reviewer (J.C.) reviewed all articles to identify and resolve discrepancies in reviewer-extracted data using the consensus feature within Covidence (individual reviewer data extraction fields are compared using the software for consistency).

### Data analysis

We exported results to Microsoft Excel for analysis and calculated basic descriptive statistics of extraction variables and percent distribution of PROGRESS-Plus categories. We then extracted themes within each PROGRESS-Plus category. Article topics were categorized into PROGRESS-Plus categories. Single-race or single-gender articles were not included in frequency counts for “race” or “gender” categories. For example, breast cancer studies of only women did not count toward the “gender” category, and studies of only AI/AN populations (with no other racial/ethnic subgroup designations) did not count toward the “race” category.

We created an evidence map using defined cancer outcomes within each PROGRESS-Plus category. The evidence map shows the density of articles (<10 articles, 10–29 articles, 30–49 articles, 50–99 articles, and 100+ articles) in each cross-section. We calculated median and interquartile ranges to examine each PROGESS-Plus category by cancer site. We did not attempt meta-analysis because of heterogeneity in study design, population, and outcome measures.

## Results

**FIG. 1.**
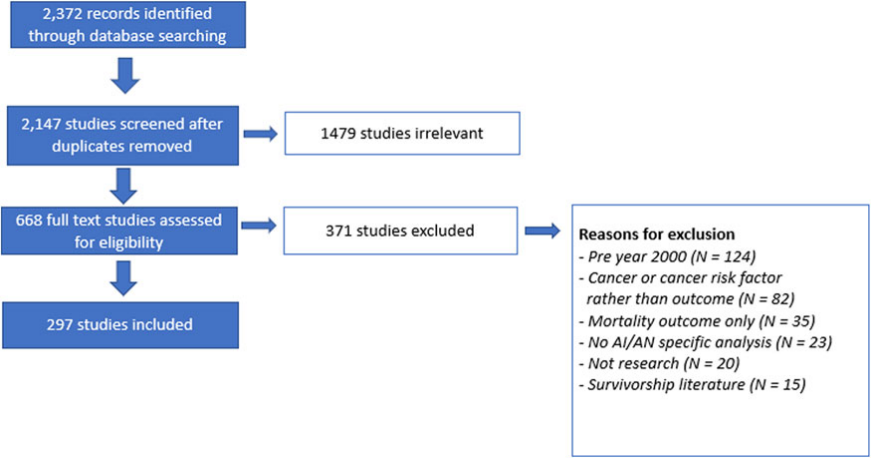
PRISMA flow diagram, articles related to SDOH and cancer risk in AI/AN populations, Published 2000–2020. AI/AN, American Indian and Alaska Native; PRISMA, Preferred Reporting Items for Systematic Reviews and Meta-Analyses; SDOH, social determinants of health.

The PRISMA diagram is shown in Figure 1. A total of 2372 studies on SDOH and cancer among AI/AN populations were imported for screening. After duplicates were removed, 2147 study abstracts were screened for eligibility. During this step, we determined that 1479 studies were out of scope. We conducted a full-text review on the remaining 668 studies, of which 371 did not meet the inclusion criteria. A total of 297 studies were included.

Descriptive statistics of the 297 articles in the final dataset are shown in Table 1. Most (93.9%) articles were observational and used a cross-sectional study design (83.2%). These studies primarily focused on the entire United States (53.9%) or on a specific state, city, or geographic area (31.6%). Few presented data for a specific tribe or tribes (4.4%). Over 60% of the articles fell into the primary category of cancer occurrence and surveillance research, and over 30% combined all cancer sites or multiple cancer sites. Articles related to specific cancers focused on either surveillance or screening for breast (17.5%), cervical (9.4%), or colorectal cancer (12.8%). Nearly 71% of the articles included no cancer risk factor other than age, sex, or race.

**Table 1.** Descriptive Statistics of 297 Articles Included in Evidence Map of Social Determinants of Health and Cancer Risk in American Indian and Alaska Native Populations, 2000–2020

Study characteristics	***N***	%
Type of study		
Intervention/experimental	18	6.10
Observational	279	93.90
Study design		
Case–control	6	2.00
Cohort	25	8.42
Cross-sectional or ecologic (includes incidence and prevalence)	248	83.50
Mixed methods	4	1.35
Nonrandomized experimental	7	2.36
Other^a^	7	2.36
Geographic reach		
Multiple countries, including United States	1	0.30
Other	2	0.70
Regional (more than one state)	26	8.80
Specific state, city, or geographic area	94	31.60
Specific tribe/tribes	13	4.40
United States	160	53.90
Unspecified	1	0.30
Study aim/interest		
Cancer occurrence and surveillance	184	62.00
Early detection/screening	81	27.30
Etiology/risk factors	20	6.70
Screening interventions	12	4.00
Sex		
Both	178	59.90
Female	103	34.70
Male	14	4.70
Not reported	2	0.70
Cancer type		
Multiple cancer sites	56	18.90
Breast cancer	52	17.50
Colon and rectal cancer	38	12.80
All cancer sites	36	12.10
Other	34	11.50
Cervical cancer	28	9.40
Lung cancer	9	3.00
Liver cancer	8	2.70
Thyroid cancer	6	2.00
Prostate cancer	5	1.70
Stomach cancer	5	1.70
Endometrial cancer (includes uterine)	4	1.30
Melanoma	4	1.30
Kidney cancer	3	1.00
Pancreatic cancer	3	1.00
Skin cancer	3	1.00
Esophageal/esophageal cancer	2	0.70
Leukemia	1	0.30
Race stratification		
AI/AN to non-AI/AN populations	225	75.76
Within AI/AN population, by IHS region	4	1.35
Within AI/AN population, by tribe	11	3.70
Within AI/AN population, with other AI/AN group(s)	7	2.36
Other^b^	11	3.70
Blank (no stratification)	39	13.13
Risk factor information		
Article includes at least one risk factor	87	29.30
No risk factor information	210	70.70
Specific risk factors included^c^		
Alcohol	23	8
Diabetes	20	7
Environmental/occupational exposure (including chemicals, air pollution, and water)	8	3
Food/nutrition (includes sugar-sweetened beverages)	16	5
Hypertension	8	3
Infectious disease (*Helicobacter Pylori*, HPV, and viral hepatitis)	19	6
Obesity/weight	34	11
Other chronic diseases	18	6
Physical activity	22	7
Reproductive/sexual health	12	4
Sun exposure/tanning	2	1
Tobacco	53	18
No. of articles, including historical context and/or discussion of historical trauma		
Yes, both historical and current trauma	12	4.04
Yes, current trauma	3	1.01
Yes, historical context OR historical trauma	22	7.41
No mention	260	87.54

All values presented as *N* or %; risk factors discussed show total *N* of risk factors in all articles; some articles have duplicate entries.

^a^ More complete list of categories provided in Supplementary Data. Examples of the “other” category include surgery studies and randomized experimental designs.

^b^ “Other” category includes less common categorizations for analysis, including counties with dense AI/AN populations, indigenous compared to nonindigenous populations, and AI/AN populations by blood quantum.

^c^ Percentages for this category do not sum to 100 because some articles included more than one risk factor group and other articles did not include any information about the listed risk factors.

AI/AN, American Indian and Alaska Native; IHS, Indian Health Service.

**FIG. 2.**
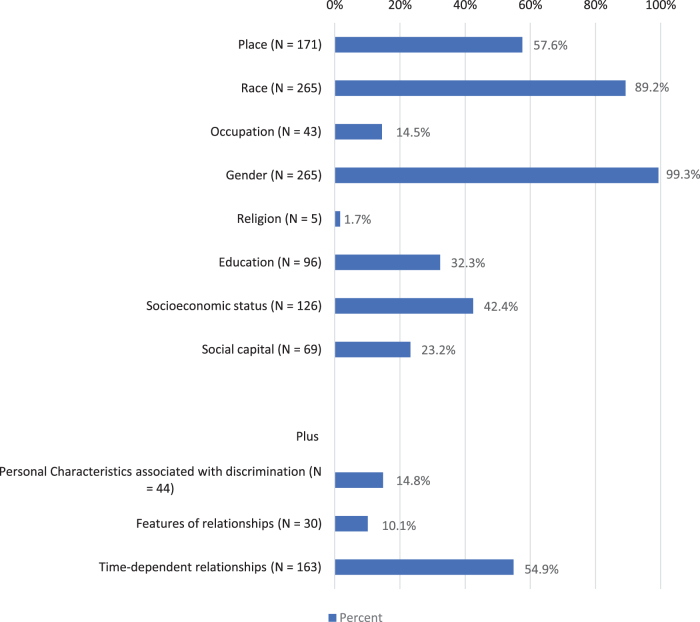
Percentage of articles about SDOH and cancer in AI/AN populations reporting PROGRESS-Plus categories (*N*=297). Articles may have multiple topics from Progress-PLUS categories; therefore, categories are not mutually exclusive.

Figure 2 identifies the distribution of PROGRESS-Plus categories. Gender (99.3%), race (89.2%), and place (57.6%) were the most frequently identified equity-related variables. Inclusion of gender variables was limited to sex-specific differences in cancer incidence rates, except one article that included nonbinary gender. Most research compared AI/AN race to other racial/ethnic subgroups (75.8%), a smaller proportion of articles (7.4%) stratified within AI/AN populations, and most of the remaining articles did not include a stratification. Social capital, education, SES, and time-dependent relationships were variable categories in 23–55% of all articles.

The least frequent equity-related variables were characteristics associated with discrimination (14.8%), features of relationships (10.1%), and religion (1.7%). Examples of themes extracted in each PROGRESS-Plus category are shown in Table 2. Supplementary Figure S1 shows the median and interquartile range for the number of PROGRESS-Plus categories and the subset of articles focusing on the most common cancer types in the study set. The median number of PROGRESS-Plus categories for each outcome ranged between three and five for all articles. These results were consistent for the articles representing the top five most frequently discussed cancer sites (*n*=216). This signals minimal variation in the number of health equity variables across outcome or cancer types.

**Table 2.** Common Themes from Data Extraction of PROGRESS-Plus Categories

PROGRESS-Plus categories	Common themes
Place	Urban versus rural, IHS region, region, state, state versus United States, county, distance/travel time, CHSDA versus non-CHSDA, rurality
Race	AIAN to non AIAN populations, AIAN population by tribe, language spoken, tribal enrollment
Occupation	Employment status (employed, unemployed, underemployed)
Gender	Male versus female cancer incidence rates, nonbinary gender (*N*=1)
Religion	Religious affiliation, church attendance, spirituality
Education	Level of education, family educational level
SES	Income, poverty, insurance status, Medicaid enrollment
Social capital	Marital status, family support, household size
Time-dependent Relationships	Age at diagnosis, stage at diagnosis
Features of relationships	Family history of cancer
Personal characteristics associated with discrimination	Disability, comorbidities/chronic disease, mental health, time spent in the United States, languages spoken

Themes represent most common subcategories assessed during data extraction, but not inclusive of all findings.

SES, socioeconomic status.

**FIG. 3.**
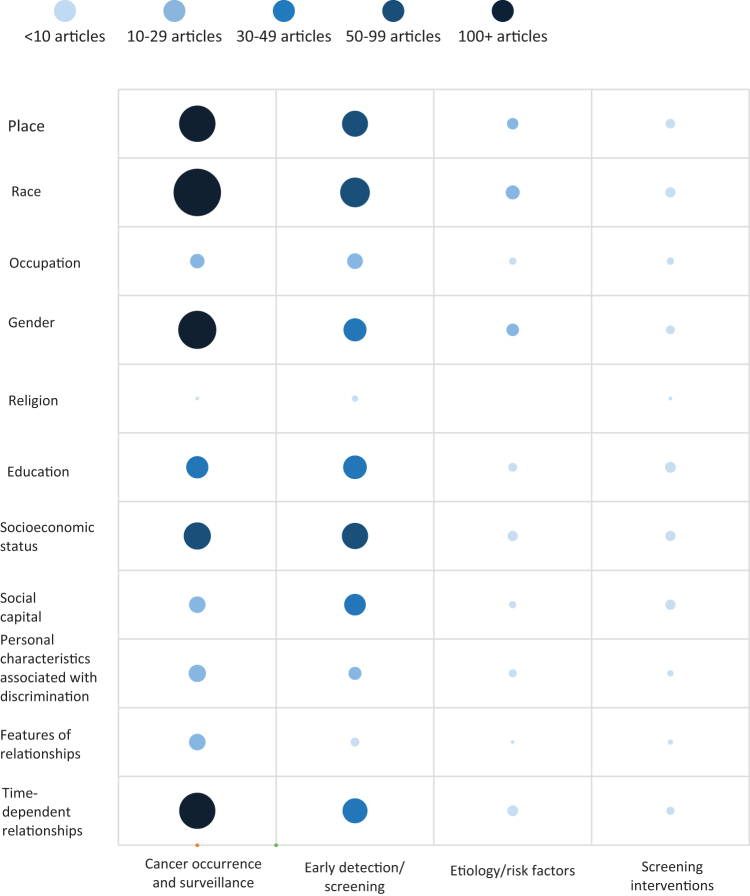
Evidence map of SDOH and cancer in AI/AN populations, by PROGRESS-Plus categories and outcomes. The evidence map and size of circles show density of articles (<10 articles, 10–29 articles, 30–49 articles, 50–99 articles, and 100+ articles) in each cross-section.

Figure 3 shows the body of evidence according to PROGRESS-Plus categories and research outcomes categories created for this review. The category for cancer occurrence and surveillance was the most represented with ≥100 articles each for place, race, gender, and time-dependent relationship. Early detection/screening research had 50–99 articles each for place, race, and SES. The etiology/risk factors and interventions categories had fewer than 10 articles in each PROGRESS-Plus category. Articles with religion or features of relationships (i.e., family history of cancer) were rare across all outcomes categories.

Out of 297 articles, only 37 (12.5%) mention historical context, historical trauma, or current trauma in the introduction or discussion (Supplementary Table S1). Of those articles, 8% include mention of IRBs or tribal reviews of research. In total, 137 articles (46%) mention IRBs or tribal review (Supplementary Table S1).

## Discussion

The purpose of this review was to create an evidence map to characterize the current state of published research on SDOH and cancer occurrence and screening among AI/AN populations in the United States. The authors are unaware of published reviews (scoping, systematic, or integrative) on this subject for AI/AN populations. Out of nearly 2400 articles identified, 297 articles published since the year 2000 fit our inclusion criteria. Most of these articles investigated disparities in cancer incidence and prevalence and focused mainly on epidemiologic data from the central cancer registries. Using the PROGRESS-Plus framework, we found that a large proportion of these articles, a majority of which utilize cancer registry data, focus on gender, race, or place. Because a large proportion of articles in this review utilized registry data, fewer included variables not routinely collected by most registries such as occupation, education, discrimination, or religion.

### Representation of race

This review revealed limitations in the current state of cancer prevention research with respect to SDOH. Because AI/AN race was the primary focus of this review, a race variable was expected to be well represented. Nearly 76% of articles in our evidence map compared AI/AN populations to other racial or ethnic subgroups, primarily White populations. Few examined cancer disparities across AI/AN populations by tribal nation or geography. Previous research has shown that data aggregated across AI/AN populations may not accurately represent health disparities, given the heterogeneity of AI/AN tribal nations in the United States.^7^ Furthermore, according to Tishkoff and Kidd, geography may account for more variation than racial groupings.^35^

There are notable sociodemographic differences between people who identify as AI/AN alone versus people who identify as AI/AN in combination with another race. Goins et al report that older multiracial AI/AN persons are less likely to live on tribal lands and more likely to reside in metropolitan areas.^16^ According to DeWeaver, persons who identify as AI/AN alone and live on tribal lands are the most socioeconomically disadvantaged of all AI/AN ancestral subgroups (N. DeWeaver, 2013, April 24. “Who Counts as Indian in the Census: The Multi-Racial Difference,” Unpublished manuscript).

Nevertheless, urban-dwelling AI/AN persons, who make up more than 70% of AI/AN populations, experience significant health disparities compared to the general US population and have higher poverty rates, which affect health.^36^ According to a 2021 report from the Urban Indian Health Institute, AI/AN children, families, and individuals were 2.3 to 3.8 times more likely to live in poverty than non-Hispanic White people.^36^

### Place

Articles in this review that incorporated “place” variable included themes such as urban compared to rural populations, as well as distance and travel time to health care or geographic region (see Table 2). Geographic barriers in access to health care and cancer screening have been well documented for AI/AN populations.^37–39^ While often attributed to the geographically remote locations of most reservations, geographic barriers are also represented by inequity in resources, uneven economic development, and the marginalization of certain populations.^40^

Place can also affect health through several mechanisms, including housing, infrastructure, residential segregation, public transportation, and environmental factors,^13,37,41^ which are largely absent as study variables from the research in this review. Their absence highlights a gap in our current discussions and understanding of cancer disparities. Although characteristics such as race and place have been explored in the literature, we understand far less about the significant intersection of place, SES, built environment, and race.^42^

By identifying geographic areas of particular need, data disaggregation could play an essential part in developing effective policies and programmatic initiatives to improve health and lower cancer risk across AI/AN populations.^43^ However, while disaggregating data and reporting data by tribal nation, geography, or other demographic characteristics may be most informative, challenges remain with racial misclassification in data,^7^ data availability, data quality, and lack of patient and community trust.^43^

### SES and access to care

Availability and accessibility of preventive and other health care services are limited not only by geography^44^ but also by financial or other barriers.^45^ SES variables appear with greater frequency in the articles in this review (42.4%) than specifically education (32.3%) and occupation (14.5%) variables. Themes related to SES, including individual-level poverty, income, and insurance status, are most common. Gaps exist, however, in the discussion of population- and community-level SES factors (e.g., percentage of the population living at the poverty level or median housing prices), which may help describe the impact of neighborhood environment on health disparities.^46^

### Risk factors and personal characteristics associated with discrimination

Only 29% of the research studies examined in this review incorporated measures of cancer risk factors. While these risk factors alone are not SDOH, they may be downstream consequences of SDOH. AI/AN populations experience some of the highest prevalence of common cancer risk factors.^18,37,47^ Cancer risk factors often have been framed in terms of personal choice and responsibility.^48^ SDOH frameworks, on the other hand, acknowledge the role of diverse upstream factors, such as the social and physical environment, educational access and quality, or workforce policy. Forty-four articles in this review incorporated variables related to personal characteristics associated with discrimination (i.e., disability, sexual orientation, etc.). Future study of the impact of those complex drivers of cancer disparities could fill a significant knowledge gap for cancer prevention strategies.

### Historical and current traumas

In working toward improving health disparities for AI/AN populations, researchers should work to fully understand and address SDOH (and structural inequities). Cancer prevention research among AI/AN populations must be done within the historical context of their lived experience.^3^ Yet, historical trauma, current trauma, or mention of historical context were discussed in only 12% (*n*=37) of the articles included in the evidence map. One study examined the relationship between American Indian boarding school attendance and chronic health conditions, including cancer.^4^ The psychosocial effects from historical and current trauma that affect individual, familial, and community well-being are well recognized.^14^

Moreover, it has been suggested that the effects of trauma can be passed down through generations through epigenetics or the alteration of DNA expression.^17,49^ Historical trauma is not a discrete part of the PROGRESS-Plus framework, highlighting a need to revise existing tools used to evaluate health equity issues relevant for AI/AN populations. Recognizing historical and current traumas as well as community resilience that is rooted in assets such as cultural practices, traditional ways of living, ceremony, and collective successes can guide future research.^14,50^

### More on data

Cancer surveillance data rely on patient-level data and have limited information on the community-level social determinants that drive cancer disparities.^51^ Inadequate data, small sample sizes, data aggregation and sharing practices,^52^ and lack of specificity by tribe, geographic region, or other vital characteristics also contribute to the challenges in assessing health disparities for AI/AN populations.

Some existing data sources, such as the Social Deprivation Index,^53^ the Social Vulnerability Index,^54^ and the Yost index,^55^ are composite scores of area-level deprivation or vulnerabilities (e.g., poverty or lack of access to transportation). These data sources can potentially improve our understanding of community-level factors associated with high cancer incidence rates in some geographic regions. Improved data systems and linkage methodologies could be used to integrate SDOH data and improve cancer surveillance data for AI/AN populations.^56^ Authentic engagement of AI/AN people and communities in the research process through methods such as community-based participatory research could support the collection of improved high-quality data for AI/AN populations.^57^

### Limitations

Several limitations should be acknowledged. First, while useful for a general framing of health equity issues, the PROGRESS-Plus framework does not explicitly include historical trauma and context. Second, this review only included articles with cancer or cancer screening as outcomes; many excluded studies focused on common risk factors or interventions for chronic disease that could affect cancer risk. Third, selected search terms may not have identified all articles related to SDOH and cancer inclusive of AI/AN populations. In addition, relevant studies from tribal health journals may have been missed due to the databases used for this study.

Fourth, many of the articles used cancer surveillance data, which by design are meant to monitor health outcomes and processes, but were not designed to address complex research questions. Fifth, qualitative studies were not included. Qualitative studies that employ accepted, culturally grounded research methods could provide valuable insights into cancer prevention and control efforts for AI/AN populations.^58^ In addition, current public health research methods do not account for the cultural inter-tribal variations of AI/AN populations. Finally, article selection and inclusion and exclusion criteria may have been affected by hidden biases among the researchers.

## Conclusions and Future Directions

This review revealed large gaps in research on the potential contribution of social determinants as drivers of cancer disparities among AI/AN populations. Research is lacking to define the causal pathways from systems, policies, and practices through SDOH to cancer risk. This includes clearly articulating the role of SDOH in cancer-related disparities and cancer risk among AI/AN populations.

Efforts to incorporate understudied topics (i.e., discrimination, racism, occupation, and education) related to the underlying contributors to health inequities can help to further characterize the impact of SDOH on cancer risk among AI/AN populations. Future research may prioritize the development of new intervention strategies to reduce cancer risk and explore historical and cultural factors to provide context for cancer-related outcomes. Future work may also aim to include studies from tribal health journals and incorporate different analytic methodologies, such as topic modeling analysis, to better understand the context of existing research in this area. In addition, a future review that systematically addresses the rich body of qualitative research in SDOH and cancer in AI/AN populations would be of value.

This review highlights gaps in current methodological frameworks for assessing health equity science. Historical trauma and historical context are not an explicit part of the framework used in this review. Both are recognized as important contextual factors related to health disparities among AI/AN populations.^3,14^ Therefore, future work could focus on developing and evaluating a health equity framework that explicitly includes these factors.

Dismantling SDOH-related disparities in cancer risk among AI/AN populations is a complex task that will require innovation, coordination, and collaboration of resources across multiple disciplines, including authentic engagement of AI/AN communities and researchers. None of the authors of this review identify as an AI/AN person. Given the scarcity of indigenous-identifying researchers in the United States, it is possible that the included research projects were the result of research agendas set and implemented by non-AI/AN persons.

A “business as usual” research agenda—one that focuses simply on individual-level risk factors—is unlikely to identify valid causal predictors of cancer risk or contribute to marked improvements in health for AI/AN populations. Models for addressing and studying SDOH in AI/AN communities should consider the culture and context of tribal populations and use approaches that take into account the strengths of these communities.^59^ Identifying gaps in knowledge and research in SDOH and cancer risk in AI/AN communities is the first step toward achieving health equity.

## Supplementary Material

Supplemental data

Supplemental data

Supplemental data

## Abbreviations Used

AI/AN: American Indian and Alaska Native
IHS: Indian Health Service
IRB: Institutional Review Board
PRISMA: Preferred Reporting Items for Systematic Reviews and Meta-Analyses
SDOH: social determinants of health
SES: socioeconomic status
