# Supplementary material for: Social Determinants of Cancer Risk Among American Indian and Alaska Native Populations: An Evidence Review and Map
Source: Health Equity. 2022 Sep 21;6(1):717–28. doi: 10.1089/heq.2022.0097 (PMC9536331; doi:10.1089/heq.2022.0097)
Supplement: Supplemental data [file Suppl_Data.docx]

**Supplement A Materials**

# Sample Search String for OVID

(age OR gender OR education OR educat* OR income OR wealth OR ethnic OR ethnic* OR race OR culture OR language OR occupation OR religion OR social class OR socioeconomic OR health social determinants OR social determinant* OR social capital OR residence OR medical geography OR health service OR health service* OR health equity OR disparit* OR medical sociology OR prejudice OR health insurance OR health gradient OR health gap OR vulnerable populations OR ethnic groups OR social conditions OR urban OR rural OR urban health OR urban population OR rural health OR rural population OR social position OR poverty OR wealth OR rich OR poor OR social support OR discriminat* OR differenti* OR urbanization OR urbaniz* OR urbanis* OR food insecurity OR food OR built environment OR environment OR Social Connectedness OR social OR Community connectedness OR community OR clinical access OR access OR Housing Insecurity OR hous* OR Immigration OR immigrant* OR Transport* OR neighborhood disadvantage OR neighborhood OR immigration OR socio-economic OR economic OR profession* OR Inequ* OR employ* OR depriv* OR Trauma OR Abuse OR hist* OR language OR family structure OR fam* OR tradition* OR institution* OR rac* OR stress OR work* OR geography* OR policy OR adverse childhood OR literacy OR violen* OR suicide OR homicide OR water OR epigen*) AND (cancer.mp. or exp Neoplasms/) AND (American Indian* OR Native American* OR exp Indians, North American/)

# Data Extraction Fields in Covidence

1. Study Design 1

- Intervention/ Experimental
- Observational

1. Study Design 2

- Randomized control trial
- Non-randomized experimental
- Cohort study
- Case control
- Systemic review (should be excluded)
- Qualitative research (should be excluded)
- Cross sectional studies or ecologic e.g., prevalence study, incidence, surveys, etc.
- Case report or case series (likely excluded due to small sample)
- Mixed methods (report on quantitative findings only for extraction)
- Other

1. Total Number of Study Participants (not just AI/AN)
2. Study Aim/Interest

Categories selected from the International Cancer Research Partnership (ICRP) Common Scientific Outline or 'CSO', a classification system organized into six broad areas of scientific interest in cancer research. <https://www.icrpartnership.org/cso>

- Biology (should be excluded, generally)
- Etiology (causes of cancer) (includes exogenous factors on origin and cause including: lifestyle factors, social determinants, nutrients, environment, infectious agents, contextual factors (race, community factors) on origin, cause, increasing risk and relationship to cancer incidence and mortality or connection to incidence or mortality; epigenetics of viruses that cause cancer; MUST have cancer-related variable to be included not just risk factor)
- Prevention Intervention (individual and population-based primary prevention interventions, which reduce cancer risk by reducing exposure to cancer risks and increasing protective factors) (personal behaviors such as physical activity, sun exposure, tobacco; dietary interventions to reduce cancer risk; chemoprevention; vaccines; complementary prevention approaches; resources and infrastructure for prevention; MUST have cancer-related variable to be included
- Early Detection, Diagnosis, and Prognosis (staging at diagnosis studies (outcome) and screening studies (not paired with other variables/study aims/outcomes)
- Treatment (should be excluded)
- Cancer Control and Surveillance Research (broad range: tracking cancer cases (surveillance); beliefs and attitudes that affect behavior regarding cancer control; ethics; education and communication approaches for patients, family/caregivers, and health care professionals; and health care delivery in terms of quality and cost effectiveness)

1. If the study variable(s) included one Risk Factor, please select from list below if only ONE, otherwise use cut and paste from this list into Risk factor2

- Adverse Childhood Events
- Alcohol
- Diabetes
- Hypertension
- Other Chronic Diseases
- Environmental/ Occupational exposure (including chemicals, air pollution, water)
- Food/ Nutrition (incl sugar sweetened beverages)
- Infectious Disease (H-pylori, HPV, viral hepatitis)
- Obesity/ Weight
- Physical Activity
- Sun Exposure/ Tanning
- Tobacco

1. Risk factor2 (copy and paste text from list above)
2. Key Findings/Summary
3. Geographical Reach of the Study (Progress +)

- Multiple Countries Including the United States
- United States
- Regional (more than one state)
- Specific State, City or Geographic Area
- Specific tribe or tribes
- Other

1. Study setting (not variable) (use these terms in the description of the study setting or population)

- Urban population/ setting
- Rural population/ setting
- Urban vs rural population/ setting
- Unspecified (no mention of urban and/or rural)
- Other

1. If Other or a Specific Geographic Area, List Below
2. Place - Tribal Affiliation(s) (if available) (Progress +)
3. Gender (Progress +)

- Male
- Female
- Both
- Not reported
- Other

1. Age(s) of Study Participants (range in study; or inclusion criteria for participation): (Progress +)

# PROGRESS - Plus Characteristics For more information see: https://methods.cochrane.org/equity/projects/evidence-equity/progress-plus

1. Was place a variable in the study?
2. If yes, what other variables related to place did the study address? (e.g., state, distance, travel time, urban v rural, reservation v non-geographic barriers to access, safe environment)
3. Was race a variable in the study?
4. Did the study include stratifications by race, ethnicity, tribal affiliation, or Indian Health Service region?
5. If findings were stratified, how

- Within AIAN population, by tribe
- Within AIAN population, by IHS region
- Within AIAN population, with other AIAN group(s)
- AIAN to non-AIAN populations
- Other

1. Was some other aspect of race/ethnicity/culture/language a variable in the study besides race?
2. If yes, what other variables related to race/ethnicity/culture/language did the study address? (e.g., historical trauma, forced removal from lands, abuse, loss of language and culture, language spoken, institutional inequities/ social inequities - racism, toxic stress, community and individual trauma, tribal and state relations)
3. Was occupation a variable in the study?
4. If yes, what variables related to occupation did the study address? (e.g., employment status, job role, unemployment, or underemployment)
5. Was gender a variable in the study?
6. If yes, what variables related to occupation did the study address other than binary gender definitions?
7. Was religion a variable in the study?
8. If yes, what variables related to religion did they study?
9. Was education a variable in the study?
10. If yes, what variables related to education did the study address? (e.g., education level, low educational attainment, limited opportunity for equal educational attainment)
11. Was socioeconomic status a variable in the study?
12. If yes, what variables related to socioeconomic status did the study address? (e.g., income, poverty, insurance status, economic exploitation, financial barriers in access to care, type of insurance, food security, housing security, FIDPR)
13. Was social capital a variable in the study?
14. If yes, what variables related to social capital did the study address? (e.g., marital status, connections, networks, support)
15. Was age a variable in the study?
16. Were other variables related to personal characteristics associated with discrimination or was discrimination variables in the study?
17. If yes, what variables related to personal characteristics associated with discrimination did the study address? (e.g., disability, sexual orientation) (e.g., disability can include mental health, multiple or significant co-morbidities, health issues significant enough to impact QOL (ESRD, symptomatic heart disease), intellectual disability, chronic pain, blindness)
18. Were features of relationships variables in the study?
19. If yes, what variables related to the features of relationships did the study address? (e.g., smoking family members, history of violence, ACEs, factors that lead to ACEs, factors that lead to violence in the home, factors associated with suicide)
20. Were time-dependent relationships a variable in the study?
21. If yes, what variables related to time-dependent relationships did the study address? (e.g., age at diagnosis, stage and diagnosis, leaving the hospital, transitions of are, respite care)
22. Did the INTRODUCTION or DISCUSSION discuss Native health outcomes/experiences in terms of historical context OR trauma or current trauma?

- Yes, historical context OR historical trauma
- Yes, current trauma
- Yes, both historical and current trauma

1. Type of cancer studied Or for screening or risk factor the intended cancer screened for:

List includes top cancer incidence for AIANs (differs from general population). See additional options for cut/paste for other at <https://www.icrpartnership.org/cancer-type-list>

- Breast Cancer
- Cervical Cancer
- Colon and Rectal Cancer
- Endometrial Cancer (includes uterine)
- Esophageal / Oesophageal Cancer
- Kidney Cancer
- Leukemia
- Liver Cancer
- Lung Cancer
- Melanoma
- Oral Cavity and Lip Cancer
- Pancreatic Cancer
- Prostate Cancer
- Skin Cancer
- Stomach Cancer
- Thyroid
- Multiple Cancer Sites
- All Cancer Sites
- For "Other" cut and paste from list here https://www.icrpartnership.org/cancer-type-list
- Other

1. Data source, e.g., name of registry, database, data source, instruments (standard or self-developed), etc.

# Exclusion Criteria

| **Exclusion Criteria** | |
| --- | --- |
| **CODE ***bold will be used for categorization purposes as well****** | **DEFINITION** |
| NEW: Year < 2000 | Articles published before the year 2000 |
| **Qualitative (tag)** | Qualitative research methods, e.g., focus groups, ethnography, etc. |
| **First Nations (tag)** | Canadian tribes |
| Other non-U.S. | Asian Indian, Chile (refer to their indigenous people as Indian), etc. |
| **Review (tag)** | Systematic, literature review, integrative review, etc. not original research |
| Clinical trial | Studies about clinical trials or clinical trials, e.g., drugs |
| Genetic study | Note: articles about genotyping of risk factors, e.g., HPV will be included |
| Non-human study | Bench science, mice, etc. |
| Survivorship literature | Cancer is not an outcome |
| NEW: Survival, mortality, or treatment as outcomes (dependent variables) | If includes incidence, prevalence, relative risk, or staging, diagnosis AND one of these three outcomes/variables will keep, otherwise exclude |
| **NEW: Risk factors for cancer without cancer or cancer screening as a direct outcome or variable** **(tag)** | E.g., tobacco cessation intervention that only references cancer but doesn’t include measures, study of incidence of smoking |
| Not research | Case study  Opinion piece  Book chapter  Methods |
| Provider focused intervention | Intervention study designed to address health care providers, e.g., Educational interventions, etc. |
| No separate AI/AN analysis | No separate analysis of AIAN group/ sample was multiracial, but results were not stratified by race |
| Insufficient sample size | TOTAL sample size <10, OK if AI/AN <6 for statistical purposes, may revisit later |
