# Supplementary material for: Social Determinants of Cancer Risk Among American Indian and Alaska Native Populations: An Evidence Review and Map
Source: Health Equity. 2022 Sep 21;6(1):717–28. doi: 10.1089/heq.2022.0097 (PMC9536331; doi:10.1089/heq.2022.0097)
Supplement: Supplemental data [file Suppl_FigS1.docx]

**Supplemental Figure 1: Median and Interquartile Range of PROGRESS-Plus Items Reported Per Review Category. A: All Articles (297); B: Articles Covering Top 5 Most Frequently Discussed Cancer Site (All Sites, Multiple Sites, Breast, Cervical, Colorectal N = 216)**

A.

B.
