# Supplementary material for: Social Determinants of Cancer Risk Among American Indian and Alaska Native Populations: An Evidence Review and Map
Source: Health Equity. 2022 Sep 21;6(1):717–28. doi: 10.1089/heq.2022.0097 (PMC9536331; doi:10.1089/heq.2022.0097)
Supplement: Supplemental data [file Suppl_TableS1.docx]

| **Supplemental Table 1: Mention of Institutional Review Board Approval Process by Historical Context/Trauma Inclusion** | | | | | | | |  |
| --- | --- | --- | --- | --- | --- | --- | --- | --- |
|  |  |  | **Yes, IRB mentioned** | | **No, IRB not mentioned** | | **Total** | |
|  |  |  | N | % | N | % | N | % |
| Was Historical or Current Trauma/Historical Context included in Introduction or Discussion | | |  |  |  |  |  |  |
|  | No |  | 113 | 38% | 147 | 49% | 260 | 88% |
|  | Both Historical and Current Trauma | | 5 | 2% | 7 | 2% | 12 | 4% |
|  | Current Trauma | | 1 | 0% | 2 | 1% | 3 | 1% |
|  | Historical Context or Trauma | | 18 | 6% | 4 | 1% | 22 | 7% |
|  |  |  |  |  |  |  |  |  |
| IRB: Institutional Review Board.  Note: If the article mentioned the IRB, even if approval from the board was not deemed necessary, it is included in the “yes” column. | | | | | | | | |
|  |  |  |  |  |  |  |  |  |
|  |  |  |  |  |  |  |  |  |
